# Association of GBA Genotype With Motor and Functional Decline in Patients With Newly Diagnosed Parkinson Disease

**DOI:** 10.1212/WNL.0000000000011411

**Published:** 2021-02-16

**Authors:** Jodi Maple-Grødem, Ingvild Dalen, Ole-Bjørn Tysnes, Angus D. Macleod, Lars Forsgren, Carl E. Counsell, Guido Alves

**Affiliations:** From The Norwegian Centre for Movement Disorders (J.M.-G., G.A.), Department of Research, Section of Biostatistics (I.D.), and Department of Neurology (G.A.), Stavanger University Hospital; Department of Chemistry, Bioscience and Environmental Engineering (J.M.-G., G.A.), University of Stavanger; Department of Neurology (O.-B.T.), Haukeland University Hospital, Bergen; Department of Clinical Medicine (O.-B.T.), University of Bergen, Norway; Institute of Applied Health Sciences (A.D.M., C.E.C.), University of Aberdeen, UK; and Department of Clinical Science, Neurosciences (L.F.), Umeå University, Sweden.

## Abstract

**Objective:**

To establish the significance of glucocerebrosidase gene (*GBA*) carrier status on motor impairment in a large cohort of patients with incident Parkinson disease (PD).

**Methods:**

Three European population-based studies followed 528 patients with PD from diagnosis. A total of 440 with genomic DNA from baseline were assessed for *GBA* variants. We evaluated motor and functional impairment annually using the Unified Parkinson’s Disease Rating Scale (UPDRS) motor and activities of daily living (ADL) sections. Differential effects of classes of *GBA* variants on disease progression were evaluated using mixed random and fixed effects models.

**Results:**

A total of 387 patients with idiopathic disease (age at baseline 70.3 ± 9.5 years; 60.2% male) and 53 *GBA* carriers (age at baseline 66.8 ± 10.1 years; 64.2% male) were included. The motor profile of the groups was clinically indistinguishable at diagnosis. *GBA* carriers showed faster annual increase in UPDRS scores measuring ADL (1.5 point per year, 95% confidence interval [CI] 1.1–2.0) and motor symptoms (2.2 points per year, 95% CI 1.3–3.1) compared to noncarriers (ADL, 1.0 point per year, 95% CI 0.9–1.1, *p* = 0.003; motor, 1.3 point per year, 95% CI 1.1–1.6, *p* = 0.007). Simulations of clinical trial designs showed that recruiting only *GBA* carriers can reduce trial size by up to 65% compared to a trial recruiting all patients with PD.

**Conclusion:**

*GBA* variants are linked to a more aggressive motor disease course over 7 years from diagnosis in patients with PD. A better understanding of PD progression in genetic subpopulations may improve disease management and has direct implications for improving the design of clinical trials.

Parkinson disease (PD) is a neurodegenerative disorder defined by the presence of motor symptoms and signs, though nonmotor symptoms are common in all stages of the disease. The disease course is typically progressive and in the advanced stages, motor impairment results in significant disability.

The glucocerebrosidase gene (*GBA*) encodes the lysosomal enzyme β-glucocerebrosidase. Mutations and polymorphisms in *GBA* are estimated to be found in up to 12% of patients with PD of European descent, and mutations in 15%–20% of Ashkenazi Jewish cases, making *GBA* the most significant genetic risk factor for PD.^1–5^ *GBA*-associated PD has been shown to manifest at a younger age and with a lower median survival time from diagnosis compared to idiopathic PD.^6–8^ Further, *GBA* mutation status is an independent risk factor for cognitive impairment.^3,5,9–11^

PD is primarily a movement disorder yet few studies have analyzed the effect of *GBA* carrier status on motor decline in early PD. In this study, we address this using longitudinal data from 3 deeply phenotyped population-based studies of incident PD in Northern Europe to comprehensively survey the progression of motor impairment in early PD. By determining the role of *GBA* variants in the evolution of motor and functional decline, we provide important new insights into the distinct clinical profile of *GBA*-associated PD, with importance for both more individualized patient care and more efficient clinical trial design.

## Methods

### Cohorts, Approvals, and Patient Consents

The participants in this study take part in the Norwegian ParkWest study, the Swedish NYPUM study, and the Scottish PINE study, 3 prospective population-based longitudinal incidence studies of PD (recruitment running between 2002 and 2009) with similar study design.^12–15^ A total of 212 patients were enrolled in the ParkWest study, 211 in the PINE study, and 182 in the NYPUM study. The consent rate to participation among incident patents was 80% in the ParkWest study, 94% in the PINE study, and 94% in the NYPUM study. The patients are under continued follow-up, and only those with a confirmed clinical or pathologic (if performed postmortem) diagnosis of PD according to the UK Brain Bank criteria at their latest or final clinical visit were included. Since enrollment, 70 had a diagnosis other than PD during follow-up. Furthermore, 57 declined genotyping, 31 have no available DNA sample or DNA was not extractable, and 7 did not consent to follow-up. The remaining 440 patients were eligible for this study and the 7-year clinical visits were complete.

### Standard Protocol Approvals, Registrations, and Patient Consents

The Western Norway Regional Committee for Medical and Health Research Ethics, the Regional Ethics Review Board in Umeå, and the Multi-Centre Research Ethics Committee for Scotland approved the respective studies. Written informed consent was obtained from all patients participating in the study (consent for research).

### Clinical Assessment

The clinical assessments have been described in detail and the same procedures were followed in each cohort.^12–15^ Briefly, a study neurologist performed general medical and neurologic examinations and semi-structured interviews at baseline to obtain medical, drug, and family history (defined as the report of a first- or second-degree relative with PD). We reassessed the patients at regular follow-up visits and offered home visits to those unable or not willing to meet at the clinic to minimize attrition bias. We used the Hoehn & Yahr (H&Y) scale to rate disease stage^16^ and the Unified Parkinson’s Disease Rating Scale (UPDRS)^17^ sections II (activities of daily living [ADL]) and III (motor examination). In addition to overall motor impairment, we assessed the severity of specific motor features of PD by the summation of the relevant UPDRS III items as follows: tremor (items 20–21), rigidity (items 22), bradykinesia (items 23–26, 31), and axial impairment (items 27–30). Assessment of nonmotor features included the Mini-Mental State Examination (MMSE). Antiparkinsonian treatment was prescribed and adjusted throughout the study by a study neurologist according to best clinical judgment. We calculated levodopa-equivalent doses (LED) in accordance with published recommendations.^18^

### Genetic Analysis

The presence of *GBA* mutations (rs76763715/N370S, rs421016/L444P, and rs781152868/Y135C) or *GBA* polymorphisms (rs2230288/E326K, rs75548401/T369M, and rs369068553/V460L) in the study population has been described in detail.^5^ Briefly, genomic DNA was extracted from peripheral blood samples collected at baseline using standard methods. A total of 188 patients of the ParkWest cohort were characterized by whole exome sequencing and 5 nonsynonymous variants were detected (N370S, T369M, E326K, V460L, and Y135C) and confirmed by sequencing.^5^ These variants were genotyped in all available samples using TaqMan single nucleotide polymorphism genotyping assay (Thermo Fisher Scientific). The L444P genotype was determined using PCR–restriction fragment length polymorphism assays (primer sequences and reaction conditions available on request) and all mutations confirmed by direct sequencing of the PCR- product.^19,20^

All amino acid substitutions are numbered excluding the 39-residue signal peptide.

### Statistical Methods

“*GBA* carriers” included all patients carrying any of the detected nonsynonymous *GBA* variants. We further split *GBA* carriers into “polymorphism carriers” (E326K, T369M, or V460L) and “deleterious carriers” (Y135C, N370S, or L444P) as described.^5^ We compared between-group differences at baseline using *t* tests, Mann-Whitney *U* tests, and χ^2^ tests as appropriate.

We applied mixed linear regression analysis to determine the association between *GBA* genotype and longitudinal motor measures assessed using repeat UPDRS ADL and motor scores. Mixed models use all available data during follow-up, can handle missing at random data, and can properly account for correlation between repeated measurements: at each visit between 94.8% and 99.8% of patients had complete UPDRS ADL or motor score data. We performed the analyses without adjustment and with adjustment for sex, study cohort, age, and duration of motor symptoms at baseline. The effect sizes were comparable after additional adjustment for LED at each visit (data not shown). The time of the repeated measures was computed in years from the baseline date. All models have fixed effect of time, as well as random intercepts, random effect of time, and first-order autoregressive residual covariance structure. Tremor and axial impairment scores were log-transformed before analysis. Plot of predictive margins was created in Stata using margins and marginsplot. As estimates of associations may be affected by dropouts due to death,^21^ we performed post hoc sensitivity analysis by joint modeling of longitudinal measures of motor function and time to death using function stjm in Stata.^22^ Whereas the longitudinal measures of UPDRS ADL or motor score and the event times were found to be related, the estimated associations and effect sizes between *GBA* carrier status and the longitudinal measures were very similar to the presented results (data not shown).

Statistical analyses were conducted using IBM SPSS Statistics (Armonk, NY) version 26.0 or Stata and 2-tailed *p* values <0.05 considered significant.

### Power Calculations for the Clinical Trial

We performed power calculations for a hypothetical clinical trial of a putative neuroprotective agent with 36 months follow-up. The first scenario assumed that all newly diagnosed patients with PD (“all-comers”) would be eligible for the trial, and the second limited trial selection to carriers of a *GBA* variant. The expected trajectories for the placebo group were set to those estimated in the present study from population-averaged models adjusted for age, sex, cohort, and disease duration (for all patients or only *GBA* carriers, as appropriate). The covariance matrices were based on separate estimates of both variance and correlation, that is, respectively 137 and 0.77 for all comers and 102 and 0.67 for *GBA* carriers. The treated individuals were assumed to experience a 50% reduction in their motor decline (i.e., the intervention halves disease progression). The required sample sizes to obtain a power of between 60% and 96% to detect such differences in slopes (i.e., the between–within subjects interaction effect) at a 5% significance level were estimated using Stata function power repeated, which incorporates F tests with Greenhouse-Geisser correction for lack of sphericity.

### Data Availability

Anonymized data are available on request by any qualified investigator for purposes of replicating procedures and results.

## Results

### Baseline Profile of PD‐GBA Carriers

**Figure 1:**
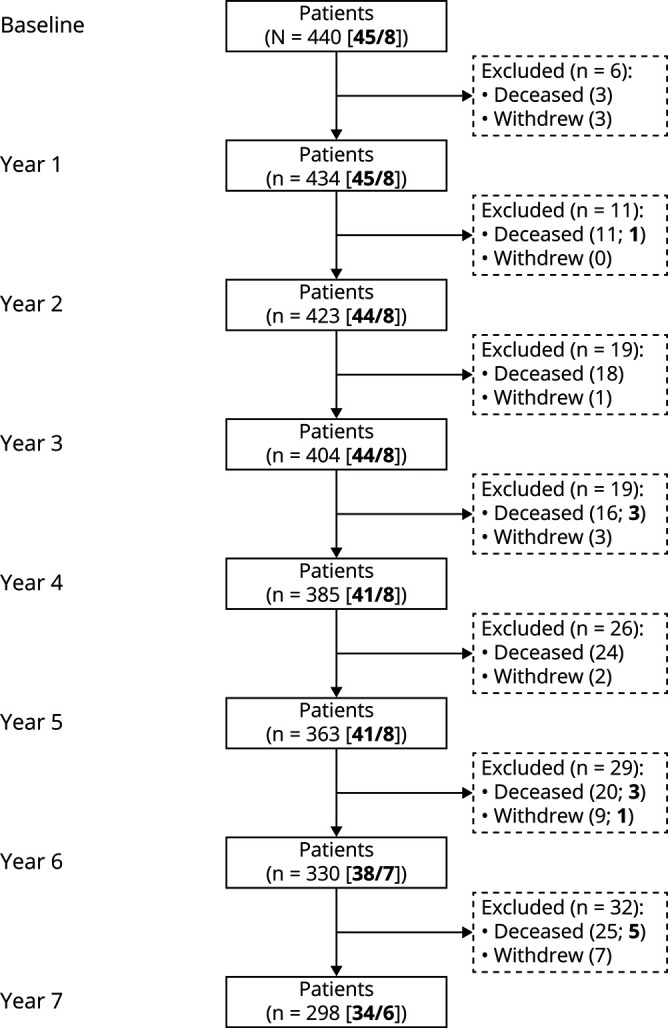
Flowchart of Study Participants Overview of patient inclusion from baseline until the 7-year visit. The number of patients in the study at each visit is shown. Withdrawals and deaths between visits are shown in dashed boxes. The number of patients carrying a *GBA* polymorphism/mutation is shown in bold. The flowchart is simplified for readability.

A total of 440 patients with PD were included in the study. Their mean age at baseline was 69.9 (±9.6) years, with 60.7% (267) male (table 1). The median duration of follow-up was 7.0 years (interquartile range 2.0). During the study, 117 patients died (26.6%) and 25 (5.7%) withdrew from the study (figure 1). *GBA* variants were identified in 53 patients: 29 E326K, 16 T369M, 6 L444P, 1 Y135C, 1 N370S, and 1 V460L variant.^5^ Carriers of any *GBA* variant observed the first motor signs of PD at an earlier age than noncarriers (*p* = 0.022) and were also younger at the age of PD diagnosis (*p* = 0.014), as previously reported.^5^ However, *GBA* carriers and noncarriers did not differ at the time of diagnosis in terms of overall motor severity or ADL function, nor severity of tremor, rigidity, bradykinesia, or axial impairment (all *p* > 0.1).

**Table 1:**
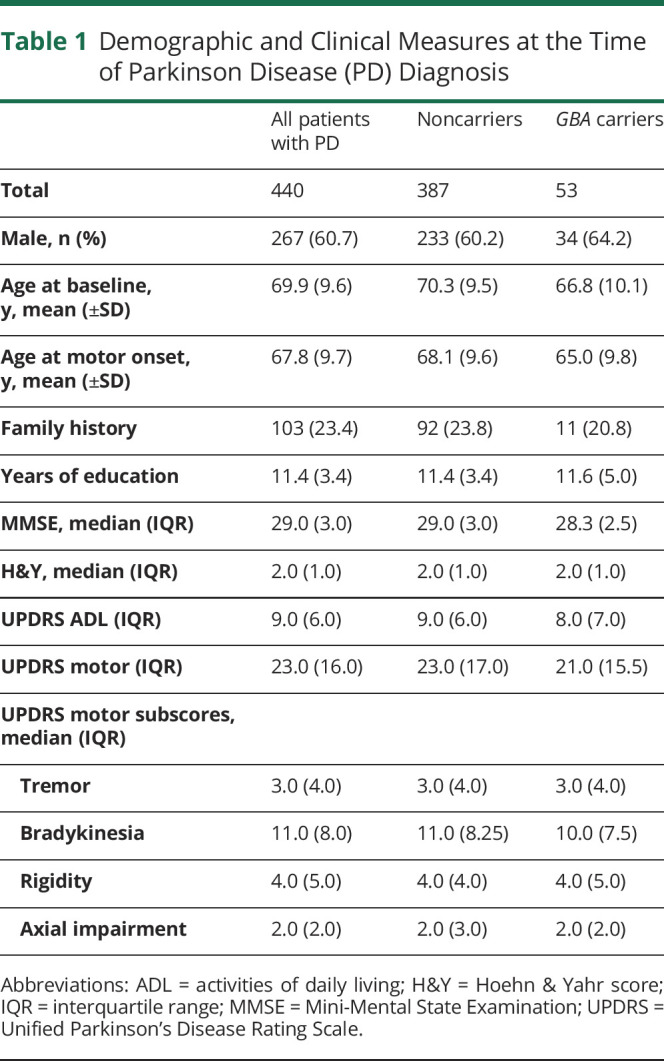
Demographic and Clinical Measures at the Time of Parkinson Disease (PD) Diagnosis

### Effect of *GBA* on Motor and Functional Decline

**Figure 2:**
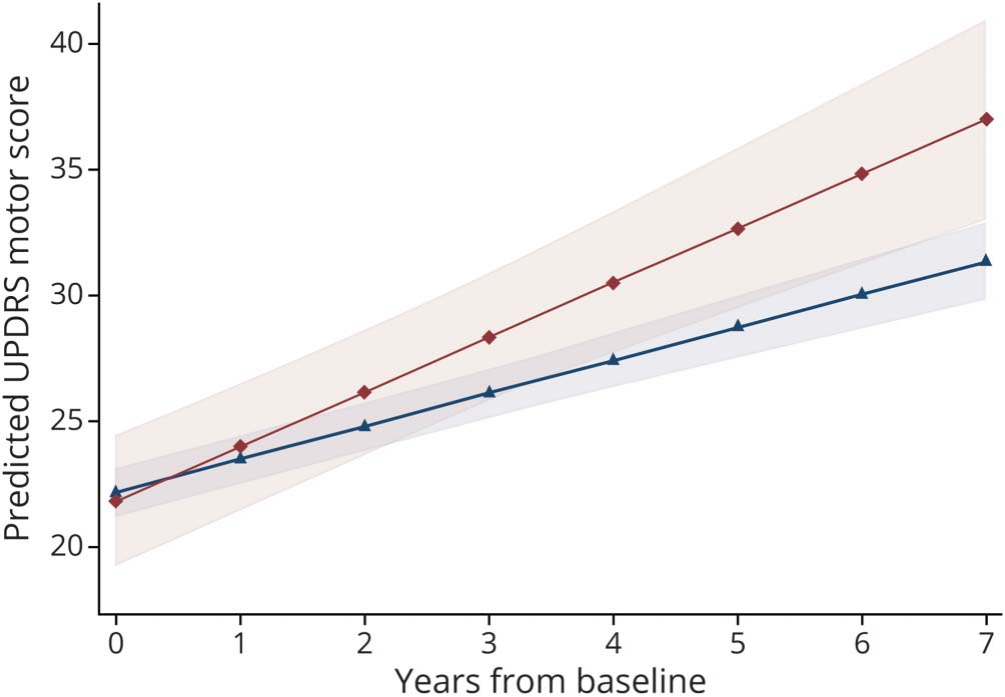
Prediction of Unified Parkinson’s Disease Rating Scale (UPDRS) Motor Score Over Time Average predicted UPDRS motor scores with confidence bands for the first 7 years after diagnosis of PD for carriers of *GBA* variants (red, diamonds) and noncarriers (blue, triangles).

Linear mixed models with adjustment for age, sex, study cohort, and duration of motor symptoms at baseline demonstrated a 69% more rapid motor decline per year in *GBA* carriers than noncarriers, with an annual increase in UPDRS motor score of 2.2 (95% CI 1.3–3.1 points) vs 1.3 point (95% CI 1.1–1.6 point), respectively. This difference was significant (0.9 point difference per year; 95% CI 0.2–1.5; *p* = 0.007) (table 2 and figure 2). We observed a similar effect on the rate of functional decline, with carriers of a *GBA* variant predicted to exhibit an annual increase in UPDRS ADL score of 1.5 point (95% CI 1.1 to 2.0), compared to only 1.0 point (95% CI 0.9–1.1 point) in noncarriers (0.5 point difference per year; 95% CI 0.2–0.9; *p* = 0.003). The effect sizes were comparable for adjusted and unadjusted models (data not shown).

**Table 2:**
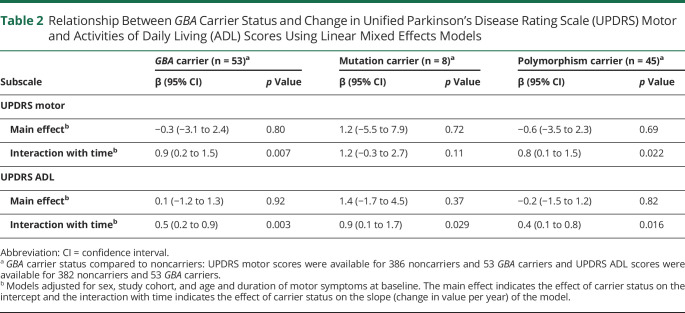
Relationship Between *GBA* Carrier Status and Change in Unified Parkinson’s Disease Rating Scale (UPDRS) Motor and Activities of Daily Living (ADL) Scores Using Linear Mixed Effects Models

Both the polymorphism and the deleterious mutation carrier groups showed faster motor and functional decline compared to noncarriers (table 2). The predicted difference in change in annual UPDRS ADL score compared to noncarriers was highest for the deleterious mutation carrier group (0.9 point difference per year; 95% CI 0.1–1.7; *p* = 0.029) and more modest for the polymorphism carrier group (0.4 point difference per year; 95% CI 0.1–0.8; *p* = 0.016). The same tendency was observed for change in UPDRS motor score per year, with a more pronounced motor decline predicted for carriers of a *GBA* mutation (1.2 point difference per year; 95% CI −0.3 to 2.7; *p* = 0.11) than a polymorphism (0.8 point difference per year; 95% CI 0.1–1.5; *p* = 0.022).

Examination of the change in specific motor symptoms revealed that the difference in motor decline was mainly driven by changes in the scores for bradykinesia and rigidity. Bradykinesia score was predicted to increase by 0.5 point per year more in carriers of a *GBA* variant compared to noncarriers (95% CI 0.2–0.8; *p* = 0.002). Further, the annual change in the bradykinesia subscore was significantly larger in carriers of a *GBA* mutation than in carriers of a *GBA* polymorphism when compared to noncarriers (table 3). Rigidity score also increased faster when comparing *GBA* carriers to noncarriers (0.2 point difference per year; 95% CI 0.0–0.3; *p* = 0.038), but no significant differences were observed for rigidity when comparing either *GBA* mutation or polymorphism carriers to noncarriers (table 3).

**Table 3:**
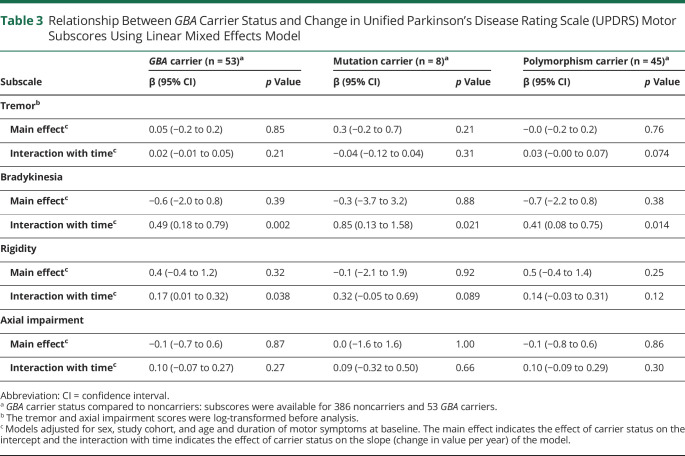
Relationship Between *GBA* Carrier Status and Change in Unified Parkinson’s Disease Rating Scale (UPDRS) Motor Subscores Using Linear Mixed Effects Model

### Hypothetical Power Analysis for a Stratified Clinical Trial Targeting *GBA*-PD

**Figure 3:**
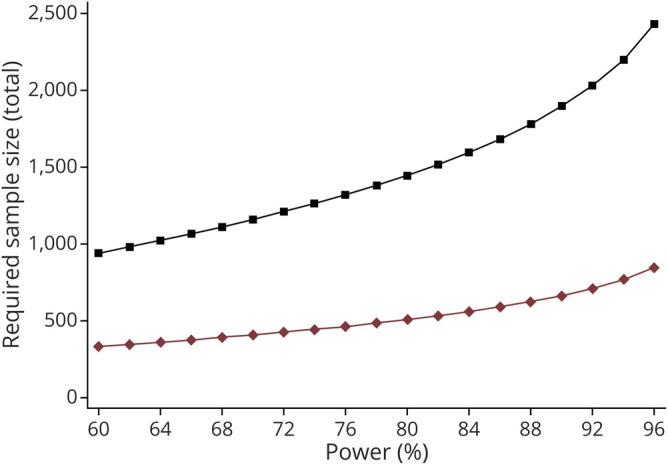
Reduced Trial Size in *GBA*-Targeted Clinical Trials Compared to the Traditional “All-Comer” Design Required sample size for clinical trials enrolling only carriers of *GBA* variants (red diamonds) or an “all-comer” design with nonselected patients with Parkinson disease (PD) (black squares) across varying levels of power to detect a between–within subjects interaction effect. A trial recruiting only PD carriers of a *GBA* variant could reduce the size required by threefold for an intervention indicated to halve the rate of motor decline, measured by the Unified Parkinson’s Disease Rating Scale (UPDRS) motor score.

Enriching a clinical trial for subgroups of patients predicted to progress faster in the primary outcome can considerably reduce trial size. To assess the reductions in trial size that could be achieved by recruiting only carriers of *GBA* variants compared to an all-comer design, we ran clinical trial power analysis for a hypothetical drug predicted to halve the progression of motor impairment (measured using UPDRS motor score). In a 3-year trial designed to have 80% power to detect between–within subjects interaction effect, we found that the required size of the trial was reduced from 1,446 participants if all newly diagnosed patients were eligible to 506 in the *GBA*-carrier design, providing a 65% reduction in the number of individuals that need to be enrolled (figure 3).

## Discussion

In this study, we comprehensively explored the relationship between *GBA* carrier status and long-term motor and functional decline in newly diagnosed patients with PD followed from time of diagnosis. Taking advantage of longitudinal data from 3 population-based studies of patients with PD in Northern Europe, our findings support a strong association between *GBA* carrier status and faster progression in motor symptoms, measured using widely used clinical and patient-reported measures of motor impairment. These data have important implications for counseling patients regarding disease prognosis and for planning and interpreting clinical trials of disease‐modifying therapies in PD.

*GBA*-PD has long been associated with a younger onset of PD and a more malignant disease course, with increased dementia risk and mortality,^3,6–11^ but studies evaluating the progression of motor impairment using the UPDRS, particularly in early PD, have been surprisingly few and inconsistent. Only 2 other studies have previously shown an association of *GBA* variants and motor impairment measured by the UPDRS, and both of these did so only in patients with more advanced disease. The first followed 13 patients with PD (average disease duration 7.5 years) and the 2 most common pathogenic *GBA* mutations (N370S or L444P) and 26 matched noncarriers, and showed more rapid motor progression in patients with *GBA*-PD.^8^ Subsequently, a large multicenter study of 733 patients (average disease duration 8.6 years) showed that carriers of a *GBA* variant experienced a larger increase in UPDRS motor score compared to noncarriers.^9^ Another study looked at the association of 2 individual *GBA* variants (N370S or K26R/rs75548401) selected from the PDgene database and progression of UPDRS motor score. No significant association was shown between either variant and the change in UPDRS motor score measured in participants from the Washington University cohort and Parkinson's Progression Markers Initiative (PPMI) cohorts using UPDRS scores in the “on” or “off” state.^23^ Of note, the low observed minor allele frequency of each variant (N370S 0.007 and K26R 0.012) combined with moderate sample size and length of follow-up of the cohorts may account for the lack of significant associations. The analysis of individual *GBA* variants may require larger collaborations; indeed the largest genome-wide association study of PD clinical phenotypes to date included 12 cohorts (including PPMI) and whereas no significant associations were identified with UPDRS motor scores, *GBA* T369M was shown to be associated with the faster development of H&Y stage 3.^24^ Alternatively, a more comprehensive assessment of *GBA* variants using exome sequencing, as done in the present study, could determine whether *GBA* carrier status affects motor impairment in these cohorts.

In our population-based study, we followed patients prospectively from the time of PD diagnosis with regular and structured clinical follow-up for up to 7 years and found carriers of a *GBA* variant on average experience a more than 69% greater annual increase in UPDRS motor score compared to noncarriers. Over the course of the study, the model predicts carriers of a *GBA* variant will experience on average a 7-point higher increase in UPDRS motor score compared to the noncarriers. This is considered to represent a moderate to large clinically important difference in motor function.^25^ In support of this, we also show substantial differences in the rate of functional decline measured by the ADL scores. Our data add an important missing piece of the puzzle, showing for the first time that carriers of *GBA* variants experience a more aggressive motor disease course from the early phases of PD.

Because *GBA* variants were associated with more rapid progression of overall motor symptoms, we further examined whether and to what extent *GBA* carrier status was associated with changes in the severity of specific motor signs. Significant differences between groups were shown in progression rates in the subscores related to bradykinesia and rigidity. It is well known that patients with PD are prone to develop dementia, with axial impairment being an established clinical risk factor.^26^ However, we found no association between *GBA* carrier status and change in severity of axial symptoms in our study. In contrast, we observed clear associations with worsening of bradykinesia and rigidity, which also have been associated with an increased dementia risk. Interestingly, while advanced age is considered a major risk factor for PD dementia,^27,28^ we and others have previously shown that *GBA* carriers develop PD at younger age than noncarriers. Overall, our data suggest that *GBA* is associated with a specific subtype of PD with more rapid motor progression and higher dementia risk despite lower age at onset and only milder axial symptoms.

We observed that the effect on changes in both the ADL and motor examination scores was faster in carriers of a *GBA* mutation compared to carriers of a *GBA* polymorphism. Similar results were observed by Davis et al.,^9^ who reported that the effect on UPDRS motor score was slightly more pronounced in carriers of pathogenic *GBA* mutations compared to carriers of the common E326K polymorphism. This observation is also in line with previous work showing that the effect size associated with *GBA* carrier status on cognitive impairment is also on a continuum.^3,9–11^ Our findings reaffirm the importance of the severity of the *GBA* variant on the progression of disease in PD.

It is established that *GBA* carriers have an earlier age at PD onset but there are conflicting reports of the presenting symptoms in *GBA*-PD compared to idiopathic forms of the disease.^29–32^ In our study, patients with PD-associated *GBA* mutations appeared clinically indistinguishable from newly diagnosed patients with idiopathic PD. This is in agreement with the only other population-based study to assess *GBA* carriers at the time of PD diagnosis, which found that patients with *GBA*-PD had a similar motor profile compared to noncarriers.^3^ Although we would not yet advocate for routine genetic testing, these data suggest that screening for *GBA* variants might be helpful as part of a model to identify those at risk of a more malignant disease course at diagnosis.

The UPDRS motor score is one of the most common outcomes in observational studies and clinical trials, used to measure the severity of PD impairment. The advantages as a primary outcome include good intrarater and inter-rater reliability but, as has been shown in this study, genetic heterogeneity in populations can substantially influence the change in UPDRS motor scores over time. We show that the *GBA*-PD subgroup, which constitutes about 12% of the PD population, are predicted to show on average 69% more rapid motor progression than the noncarriers. If not accounted for, differences in *GBA* carrier distribution by treatment arm (which can arise by chance even during randomization) could thus influence trial outcome and lead to invalid conclusions. Balancing treatment arms with stratification of randomization according to *GBA* status would avoid this potential problem in future trials.

A major challenge for drug development in neurodegenerative diseases is that adequately powered trials typically require several hundred participants and long durations. The expected progression of the trial's primary outcome in genetic subgroups can be used to improve the efficiency and effectiveness of clinical trials. The utility of this model in *GBA*-PD was assessed by comparing simulated randomized placebo-controlled trial designs to recruit all newly diagnosed patients with PD (all-comers) or only those predicted to be at risk of a faster motor decline, by limiting trial selection to carriers of a *GBA* variant. We show that a trial limited to *GBA* carriers could recruit up to 65% fewer participants than a traditional all-comer design, which could feasibly translate into considerable savings in costs. Our data are in line with and extend on previous estimations targeting only patients with a *GBA* mutation (such as L444P) for a hypothetical trial with MMSE as the primary outcome.^11^ Notably, this hypothetical trial was found to reduce trial size by as much as 25-fold compared to a trial of patients with PD without a *GBA* mutation.^11^ However, neuropathic *GBA* mutations are rare in the general PD population (1.4% in our study), whereas we considered all types of *GBA* variant and thus substantially increased the number of eligible trial participants. In light of this, the threefold reduction in trial size shown here is more realistic in a population-based setting. The first example of a trial studying ambroxol in genetically defined patients with PD was recently published,^33^ and although this trial was not powered to assess the clinical efficacy of the drug, it demonstrates the potential of using genetic information to balance or build a trial.

Our study is not without limitations. The modest sample size of each genotype group prevented the assessment of individual *GBA* mutations and larger longitudinal studies will be needed to determine whether the risk for motor progression varies across individual *GBA* variants. The use of a linear model provides an approximation of overall disease progression, as patients tend to improve during the first year after initiating medication before worsening again, and future studies should also examine possible differences in the response to medication in GBA-associated PD. Strengths of our study include the large number of participants with newly diagnosed PD with longitudinal data available for inclusion, the uniform study designs of the 3 cohorts, length of follow-up (median 7 years), the number of visits analyzed per participant (median 8 visits), and the exceptionally low attrition rate. Furthermore, each of the cohorts are population-based studies, which avoids some of the pitfalls when cases are recruited from neurology and memory clinics. This means that our findings that *GBA* variants are linked to more rapid motor progression in PD are likely to be applicable to the general PD population. Motor decline has a major bearing on independence, nursing home admission, caregiver burden, and mortality in PD,^34^ and better understanding and prediction of PD progression could improve disease management for a meaningful proportion of patients and has direct implications for improving the design of clinical trials.

## Glossary

ADL: activities of daily living
CI: confidence interval
*GBA*: glucocerebrosidase gene
H&Y: Hoehn & Yahr
LED: levodopa-equivalent dose
MMSE: Mini-Mental State Examination
PD: Parkinson disease
PPMI: Parkinson's Progression Markers Initiative
UPDRS: Unified Parkinson’s Disease Rating Scale
